# Examination of Candidate Exonic Variants for Association to Alzheimer Disease in the Amish

**DOI:** 10.1371/journal.pone.0118043

**Published:** 2015-02-10

**Authors:** Laura N. D’Aoust, Anna C. Cummings, Renee Laux, Denise Fuzzell, Laura Caywood, Lori Reinhart-Mercer, William K. Scott, Margaret A. Pericak-Vance, Jonathan L. Haines

**Affiliations:** 1 Center for Human Genetics Research, Vanderbilt University Medical Center, Nashville, TN 37232, United States of America; 2 Department of Epidemiology & Biostatistics and Institute for Computational Biology, Case Western Reserve University, Cleveland, OH 44106, United States of America; 3 Hussman Institute of Human Genomics, Miller School of Medicine, University of Miami, Miami, FL 33101, United States of America; Harvard Medical School, UNITED STATES

## Abstract

Alzheimer disease (AD) is the most common cause of dementia. As with many complex diseases, the identified variants do not explain the total expected genetic risk that is based on heritability estimates for AD. Isolated founder populations, such as the Amish, are advantageous for genetic studies as they overcome heterogeneity limitations associated with complex population studies. We determined that Amish AD cases harbored a significantly higher burden of the known risk alleles compared to Amish cognitively normal controls, but a significantly lower burden when compared to cases from a dataset of unrelated individuals. Whole-exome sequencing of a selected subset of the overall study population was used as a screening tool to identify variants located in the regions of the genome that are most likely to contribute risk. By then genotyping the top candidate variants from the known AD genes and from linkage regions implicated previous studies in the full dataset, new associations could be confirmed. The most significant result (p = 0.0012) was for *rs73938538*, a synonymous variant in *LAMA1* within the previously identified linkage peak on chromosome 18. However, this association is specific to the Amish and did not generalize when tested in a dataset of unrelated individuals. These results suggest that additional risk variation in the Amish remains to be identified and likely resides outside of the classical protein coding gene regions.

## Introduction

Alzheimer disease (AD) is the most common cause of dementia, the global loss of cognitive ability beyond the normal changes associated with aging. The prevalence of AD for individuals aged 85 years and older is 32%, and the number of people with AD is predicted to triple by 2050 [1]. The World Health Organization lists AD as the 4^th^ leading cause of death in high-income countries [2]. AD is generally categorized as early onset at age 65 or below or late onset (LOAD) after the age of 65. Despite the high prevalence and associated death, much is unknown about the cause and pathogenesis of this neurodegenerative disorder.

There are many risk factors associated with AD, including age, family history, lifestyle, activity, education, atherosclerosis and genetic factors. The heritability of LOAD is estimated at 60–80% [3]. *APOE* is the strongest genetic risk factor for LOAD, but accounts for far less than 50% of the expected genetic effects [4–13]. Large genome-wide studies have identified risk loci in or very near *CR1*, *CLU*, *PICALM*, *BIN1*, *EPHA1*, *MS4A*, *CD33*, *CD2AP*, *ABCA7*, *HLA-DRB5*/*HLA-DRB1*, *PTK2B*, *SORL1*, *SLC24A4*/*RIN3*, *DSG2*, *INPP5D*, *MEF2C*, *NME8*, *ZCWPW*1, *CELF1*, *FERMT2*, and *CASS4* [14–19]. These loci are involved in complement pathway activation, nervous system development, inflammation, synaptic transmission, and beta-amyloid regulation. However, the common variants in these loci individually confer very modest risk. Recent sequencing studies have identified rare variants in *APP* and *TREM2* with larger effect sizes [20–22]. As with many complex diseases, the identified variants do not explain the total expected genetic risk as determined through heritability estimates. The unexplained genetic risk suggests additional variants in these known genes or currently unassociated genes may confer susceptibility. Through the identification of additional risk variants or loci, more can be learned about the underlying biology and pathogenesis of AD that can inform future studies about diagnosis and treatment targets.

Most genetic studies evaluating LOAD risk are performed in general population studies, introducing analysis and interpretation problems due to heterogeneity. To further the understanding of this disease, we studied the genetically isolated Amish communities of Ohio and Indiana to identify additional genetic variants that contribute to disease risk. The population bottleneck that occurs when a small group of individuals establishes a separate subpopulation and creates a founder effect. The random drift that occurs in this new subpopulation may change disease prevalence, reduce effective population size, alter allele frequencies and change patterns of linkage disequilibrium. In particular, the Amish are more genetically homogenous because members of these communities marry within their culture, thus limiting the amount of new genetic variation introduced from the general population. Additionally, due to their strict lifestyles, environmental exposures are more homogenous. For example, the older Amish have generally led an agricultural lifestyle, achieved similar levels of education, and consumed similar diets. These factors make the Amish populations advantageous for genetic studies by further controlling for non-genetic heterogeneity.

Since these isolated populations differ from the general population, the specific variants identified through general population studies may not be present in the same frequency or have the same effect in the Amish. Conversely, since the Amish are a genetic subset of the general European Caucasian population, it is expected that the same genes and pathways implicated in the Amish will also be associated and confer risk in the general population. By studying the genetics of these isolated populations, the limitations introduced by the heterogeneity in broad population studies can be overcome and variants or genes that help explain the missing heritability of LOAD can be identified.

If the known risk alleles do not contribute the same risk in the Amish, it is hypothesized that the Amish cases will have a significantly lower burden of risk alleles when compared to the LOAD cases from a dataset of unrelated individuals. However, if all or a subset of the known genetic risk alleles do contribute to disease risk in the Amish, the Amish cases should tend to have a significantly higher genetic risk score than the Amish cognitively normal controls.

**Fig 1:**
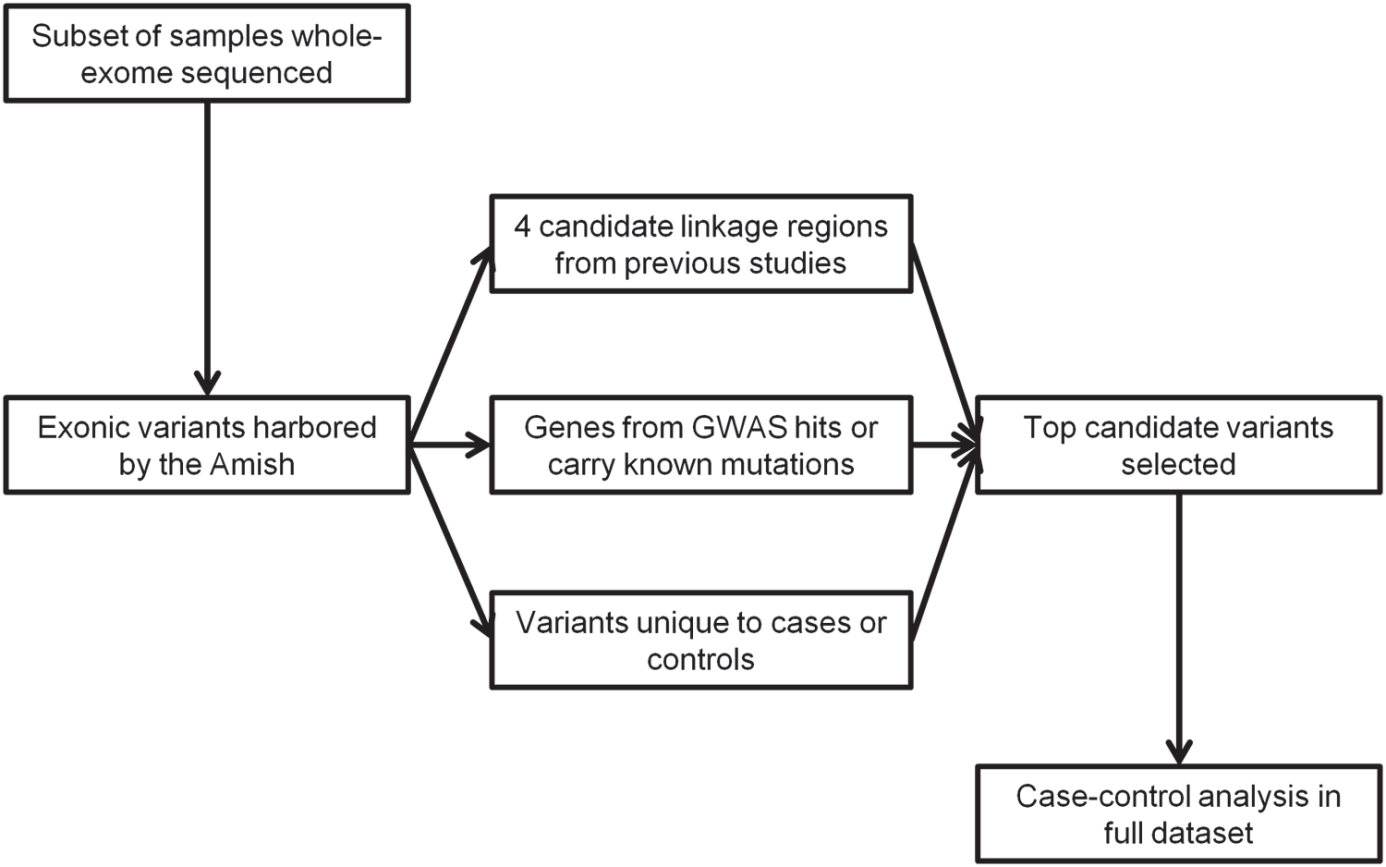
Flow Diagram of this Study. Individuals were selected from the full Amish dataset for whole-exome sequencing. The variants identified from these data were used to screen three classes of variants, genes that are very near or contain GWAS hits or carry early-onset mutations, genes within four candidate linkage regions implicated by previous studies and genes that harbor variants that occur uniquely in cases or in controls. The top variants from these three classes were then genotyped in the full dataset and case-control association was performed.

To identify additional exonic variation harbored by the Amish that may contribute to disease risk in this isolated population, this study used whole-exome sequencing of a selected subset of the overall study population as a screening tool to identify variants harbored in the regions of the genome that are most likely to contribute risk. By then genotyping the top candidate variants from this screen in the full dataset, there is more power to detect an association between the variant and phenotype of interest (Fig. 1). It is hypothesized that the top candidate exonic variants will be associated with LOAD risk in the Amish.

## Methods

### Study populations and clinical data

There were two significant waves of immigration that established the Amish communities in the United States, the first wave occurred in the 1700s with Swiss Anabaptists settling in Pennsylvania and the second in the 1800s when individuals immigrated to Ohio and Indiana from Europe and the Pennsylvanian settlements [23]. The full dataset for which samples have been collected is comprised of individuals from the Amish communities in Adams, Elkhart and LaGrange Counties in Indiana and Holmes County in Ohio. Individuals were ascertained through public directories, public notices, and referrals from previously enrolled participants. Over 30% of the Amish populations over the age of 65 have been contacted and 87% of these individuals have consented to participate in the study. The Modified-Mini Mental Status (3MS) exam was used to screen individuals during the initial interviews [24]. Information from these baseline screens and additional cognitive testing were used to generate a consensus diagnosis according to the National Institute of Neurological and Communicative Disorders and Stroke (NINCDS) and the Alzheimer’s Disease and Related Disorders Association (ADRDA) criteria [25]. Methods for ascertainment were reviewed and approved by the individual Institutional Review Boards of the respective institutions. Sample collection, DNA extraction, cognitive testing and affection statuses derived from the consensus diagnoses followed procedures detailed in previous studies conducted in these populations [26]. The full Amish dataset comprises 1,119 individuals sampled.

The Anabaptist Genealogy Database (AGDB) combined and digitized genealogy records and books that include multiple individuals and families [27]. This resource generated an all-connecting pedigree consisting of over 5000 Amish members. From this pedigree, the relationship status and degree of relatedness can be determined for all individuals in the full dataset.

As a comparison dataset for the genetic burden and variant association analyses, cases and cognitively normal controls ascertained from a general clinical population were studied [18]. A collaborative study between researchers at the University of Miami and Vanderbilt University has ascertained Caucasian individuals affected with LOAD unique from the Amish populations also studied. These individuals have been diagnosed with probable or definite AD according to NINCDS-ADRDA criteria with an age of onset greater than 60. To make these diagnoses, documentation or a clinical history of significant cognitive impairment was present. Age- and gender-matched cognitively healthy controls were ascertained from the same regions and had a documented 3MS or MMSE score in the normal range. As the Amish are founded from European immigrants, this European-American dataset of unrelated individuals is of similar ancestry.

### Ethics Statement

This research study has been continually approved by the Institutional Review Board at Vanderbilt University. This sub-committee determined the study poses minimal risk to participants and approved the Consent Form, Application for Human Research and the Protocol. Written consent was given by participants for their information and history to be used in this study.

### Analysis of genetic risk score

To determine if the known genetic etiology of LOAD also impacts LOAD in the Amish, the total genetic risk score using known LOAD risk alleles was calculated and compared across affection groups. Twenty-one SNPs that reached genome-wide significance in the most recent LOAD genome-wide association study (GWAS) were genotyped, 17 of which passed the QC for the follow-up genotyping phase, in the full Amish dataset (Table 1) [16]. Previous genotype data for *APOE* were used [26]. The weighted genetic risk score was calculated by multiplying the number of risk alleles at each marker by the weight (proportional to its published effect size) for that marker, and then summed across all markers [16]. In addition to comparing across Amish affection statuses, cases and cognitively normal controls ascertained from a general clinical population were compared (Table 2). Moreover, Amish LOAD cases were compared to unrelated LOAD cases and Amish cognitively normal controls to unrelated cognitively normal controls. Logistic regression was performed (R, version 3.0.2) to determine if total genetic risk score was correlated with affection status or population dataset.

**Table 1:** Details of Risk Loci from Meta-Analysis Used to Calculate Total Genetic Risk Score.

Marker	Chr	Position	Gene	Overall	Amish	Unrelated	Weights
				OR	MAF	MAF	
**rs6656401**	1	207692049	CR1	1.18	0.24	0.18	0.052
**rs6733839**	2	127892810	BIN1	1.22	0.45	0.4	0.062
**rs35349669**	2	234068476	INPP5D	1.08	0.45	0.5	0.024
**rs190982**	5	88223420	MEF2C	0.93	-	-	-
**rs9271192**	6	32578530	HLA-DRB5/	1.11	0.18	0.28	0.033
			HLA-DRB1				
**rs10948363**	6	47487762	CD2AP	1.1	-	-	-
**rs2718058**	7	37841534	NME8	0.93	0.29	0.35	0.023
**rs1476679**	7	100004446	ZCWPW1	0.91	0.28	0.29	0.03
**rs11771145**	7	143110762	EPHA1	0.9	0.27	0.32	0.033
**rs28834970**	8	27195121	PTK2B	1.1	0.32	0.35	0.03
**rs9331896**	8	27467686	CLU	0.86	0.36	0.41	0.047
**rs10838725**	11	47557871	CELF1	1.08	0.35	0.31	0.024
**rs983392**	11	59923508	MS4A6A	0.9	-	-	-
**rs10792832**	11	85867875	PICALM	0.87	0.45	0.35	0.044
**rs11218343**	11	121435587	SORL1	0.77	0.05	0.04	0.082
**rs17125944**	14	53400629	FERMT2	1.14	0.05	0.11	0.041
**rs10498633**	14	92926952	SLC24A4/RIN3	0.91	0.2	0.22	0.03
**rs8093731**	18	29088958	DSG2	0.73	0.01	0.01	0.099
**rs4147929**	19	1063443	ABCA7	1.15	-	-	-
**rs3865444**	19	51727962	CD33	0.94	0.29	0.3	0.019
**rs7274581**	20	55018260	CASS4	0.88	0.1	0.08	0.04
***APOE* E4**	19	19q13.2	APOE	2.5	0.14	0.26	0.287

Alleles, MAF, and overall OR are published values. Chr = chromosome. Pos = position in bp. MAF = minor allele frequency. OR = odds ratio. Adapted from Lambert, et al, 2013 [16]. Allele frequency was calculated using the 921 Amish samples and the 971 samples from the unrelated dataset that passed QC in the follow-up genotyping phase.

**Table 2:** Demographics of Genetic Risk Score Samples.

Dataset	Affection status	Female	Total	Average age of exam/onset
				(standard deviation)
Amish	**LOAD case**	63%	126	78 (7.75)
	**Cognitively normal control**	58%	503	79 (6.72)
Unrelated	**LOAD case**	63%	473	74 (8)
	**Cognitively normal control**	60%	498	74 (8)

Percent female, age of exam and onset averages and standard deviations were calculated for 629 Amish samples and the 971 samples from the unrelated dataset which passed QC for follow-up genotyping.

### Selection for sequencing

From the larger dataset of 1119 Amish individuals, 176 individuals, 59 AD cases, 68 unaffected controls and 49 unknowns, were selected for whole-exome sequencing for several different studies (LOAD, Parkinson’s disease and age-related macular degeneration). Individuals were chosen for the LOAD using the following prioritization (a) large sibships with both affected and unaffected individuals, (b) close relatives of sibships in (a), (c) *APOE* genotype, and (d) members of subpedigrees with high linkage results from previous studies [26]. We hypothesized that these 59 AD cases are most likely to harbor unidentified variants that confer risk to LOAD.

### Sample preparation and exome sequencing

Paired-end whole-exome sequencing was performed on DNA extracted from blood. Two sequencing sites, the Genome Sciences Resource at Vanderbilt University Medical Center and the sequencing core of the Center for Genomic Technology at the Hussman Institute for Human Genomics (HIHG) at the University of Miami Miller School of Medicine, performed the exome sequencing. The Agilent SureSelect Human All Exon 50 Mb capture kit was used to capture the exonic genomic DNA. This exonic library was then sequenced on the Illumina HiSeq 2000, with paired ends and read lengths of 75 base pairs.

### Processing of raw sequences and calling of variants

Sequence processing consisted of aligning reads, removing duplicates, realigning around local indels, recalibrating quality scores and calling of variants. Using BWA (version 0.6.2), raw sequences reads were aligned to the UCSC hg19 human reference genome. Picard tools (version 1.74) was used in the process of marking duplicates. All steps performed in the Genome Analysis Tool Kit (GATK, version 2.1–10) followed the best practices available at the time of processing, these consisted of local realignment around indels, base recalibration, multi-sample variant calling (using the UnifiedGenotyper), and variant recalibration. The reference bundle was downloaded from GATK and used for all processing steps.

Data management was performed using vcftools (version 0.1.9) for additional QC steps. Samples with an average depth less than 30 (n = 9), samples with a concordance rate less than 90% with previous genotyping (n = 3), and samples with discordant genders between the sequencing and the genders recorded in the clinical data (n = 2) were removed. There were a total of 170,849 variants called across the 162 whole-exomes (53 LOAD cases, 65 cognitively normal controls and 44 of unclear/unknown status) that passed the above QC measures. Of these variants, 153,272 passed the processing filter of a minimum phred-scaled quality threshold of 10. To control for missing data, variants with a calling efficiency of less than 80% were removed from analysis. Only biallelic markers were analyzed as the software used to test for association while correcting for the pedigree structure is restricted to biallelic markers. After this QC, 162 individuals and 79,203 exonic variants were analyzed (Table 3). This QCed dataset was 99.1% concordant for 8,268 exonic variants overlapping with previous genotyping and individuals were sequenced at an average depth of 58.60 ± 13.53.

**Table 3:** Demographics of Amish Exome Sequencing Samples.

Affection status	Female	Total	Average age of exam/onset
			(standard deviation)
LOAD case	55%	53	78 (6.92)
Cognitively normal	62%	65	76 (7.21)
Unclear or unknown	41%	44	78 (7.60)

Percent female, age of exam and onset averages and standard deviations were calculated for the 162 samples which passed QC for whole-exome sequencing.

### Prioritization of identified variants

To overcome low power due to small sample size in the initial screening population, variants from three classes of genes were prioritized for follow-up analysis in the full dataset. Class one included 26 genes previously implicated in LOAD through GWAS and early-onset mutations [14–19]. Class two genes resided in four previously identified candidate linkage regions in this Amish dataset [26]. Class three genes harbored variants that occurred uniquely in cases or in controls. There was no overlap between the previously implicated AD genes and genes under the four linkage peaks. A total of 56 variants (25 in AD genes, 30 in linkage regions, and 1 unique to cognitively normal controls) were identified from the sequencing data and thus were prioritized for genotyping in the full Amish dataset (S1 and S2 Tables). The criteria for prioritization were a nominally significant association p-value (< 0.01) in the sequencing data or because the variant was not present in three catalogs of human variation (dbSNP build 137, ESP 6500 release, and 1000 Genomes April 2012 release).

### Genotyping of selected variants

Fifty-four of the prioritized variants were genotyped in the full Amish dataset using three pools designed for the Sequenom iPLEX Gold assay on the MassARRAY platform. This technology is based on a single-base primer extension reaction coupled with mass spectrometry. The remaining two variants were genotyped via pre-designed TaqMan assays that contain allele-specific primers and fluorescent probes. In addition to the variants identified from the sequencing experiments, the 21 GWAS hits used for the genetic risk score analysis were genotyped in these pools in both the Amish and the unrelated datasets. Seven variants (two GWAS hits and five sequencing variants) failed genotyping. Of the remaining 70 variants, two failed to validate and thus were monomorphic in the larger dataset. Additionally, two variants with low efficiency and one multiallelic marker were dropped from analyses. One variant had low concordance with the sequencing data and genotypes were manually called from the cluster plots. This resulted in 48 sequencing variants and 17 GWAS hits passing these QC measures (Table 4).

**Table 4:** Summary of Variant QC from the Follow-up Genotyping.

	Sequence	GWAS hit	Complete
	Variant		Dataset
**Selected from sequence data**	56	21	77
**Failed to genotype**	5	2	7
**Failed to validate, monomorphic**	2	0	2
**Dropped due to low marker efficiency**	0	2	2
**Dropped due to multiallelic variant**	1	0	1
**Available for analysis**	48	17	65

Sequence variant = variant identified from whole-exome sequence data. GWAS hit = SNP implicated by two recent meta-analyses [16,18]. Complete dataset = all variants and markers genotyped in three Sequenom pools and two TaqMan assays.

A total of 1,119 unique Amish samples were genotyped for the 77 variants. Eighty-three samples were dropped due to a genotyping efficiency below 95%. Two individuals were dropped from analysis for low concordance between follow-up genotyping and the sequencing data. To calculate kinship coefficients to adjust for relatedness, individuals not currently in the AGDB and those who were not in the subsequent all-connecting pedigree were removed. This resulted in 921 samples passing all QC measures (Table 5).

**Table 5:** Demographics of Amish Samples Used For Follow-up Genotyping.

Affection status	Female	Total	Average age of exam/onset
			(standard deviation)
LOAD case	63%	126	78 (7.75)
Cognitively normal	58%	503	79 (6.72)
Unclear or unknown	49%	292	80 (6.82)

Percent female, age of exam and onset averages and standard deviations were calculated for the 921 samples which passed QC for follow-up genotyping.

### Analysis of single variant in cases versus controls

Case-control association in the Amish was performed using the Modified Quasi-Likelihood Score (MQLS) test, which corrects for the relatedness of individuals [28]. This program uses the kinship coefficient, a measure of relatedness between two individuals, to account for the pedigree structure. Additionally, this method allows for the inclusion of samples with unknown or unclear affection status, increasing the overall sample size. Type 1 error rates for the method are not inflated when used for the Amish [29]. This association test was used for both stages of the study, analysis of sequence variants and follow-up genotyping. A conservative Bonferroni correction for the number of tests performed in each stage was used to determine the threshold for the level of significance. To generalize the results of any significant association in the follow-up genotyping phase, logistic regression was performed in PLINK (version 1.07) with *APOE* as a covariate to test for association in the unrelated dataset.

### Power studies

Power is dependent on a number of variables, including sample size, allele frequency and effect size. The small sample size of the sequencing dataset (162 exomes passing QC measures) is likely to be too small even to detect an association for a common allele with a moderate effect size. For example, if 162 unrelated cases and an equal number of controls were sequenced or genotyped for a variant with a minor allele frequency of 5% and an OR of 2, the power to detect an association is only 34.7% if the type I error rate is 0.05. This estimate assumes individuals are unrelated and therefore is an overestimate of the power in this population of related individuals.

By genotyping the prioritized variants in over 1,100 samples, the power limitations of the screening population may be overcome and associations may be detected. Previous studies in this Amish population investigated this software’s power to detect associations [29]. For dominant and additive models, there was greater than 90% power to detect an association at p < 0.05 when the simulated odds ratio (OR) was at least 2 and the minor allele frequency was held constant at 0.2. For genome-wide data, the Bonferroni-corrected p-value is traditionally 5 x 10–8. If the OR is 5, there was 90% power to detect an association for dominant and additive models, but this power dropped significantly, less than 5%, if the OR was less than or equal to 2.

In the unrelated dataset, there was at least 90% probability to detect an association, if present, when the effect size was at least 1.25 with a type I error probability of 0.05. The association program used in the Amish, MQLS, does not calculate an OR or effect size for the variant being tested so this power calculation is an estimate that may vary based on the true effect size.

## Results

### Analysis of genetic risk score

**Fig 2:**
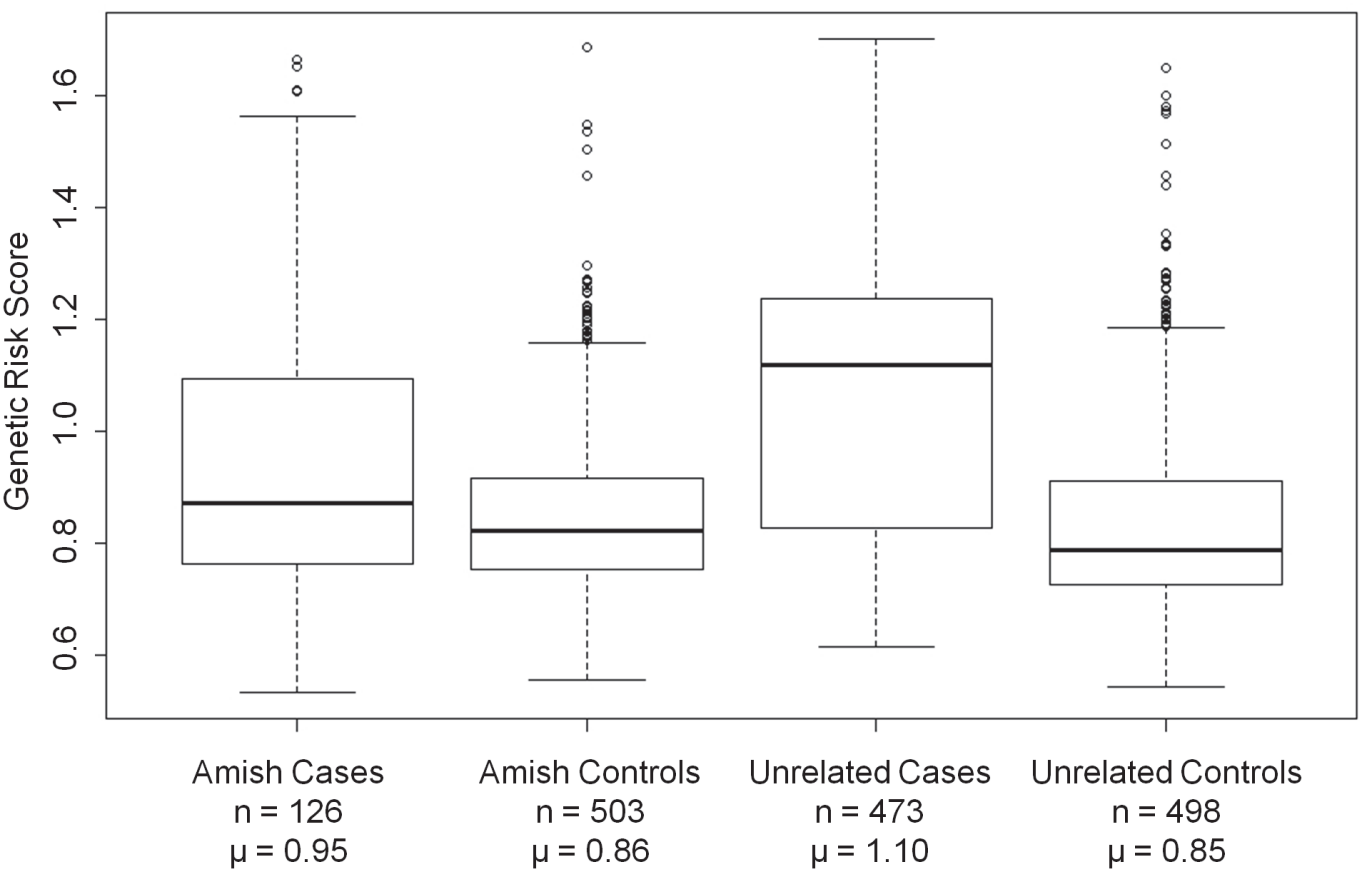
Distributions of Total Genetic Risk Scores. Total genetic risk score averages and standard deviations were calculated for the 629 Amish LOAD cases and cognitively normal controls and the 971 LOAD cases and cognitively normal controls from the unrelated case-control dataset who passed QC for the follow-up genotyping phase. n = total number of individuals. μ = average total risk score for group.

Total genetic risk score was calculated for each individual in the study population to evaluate the genetic contribution of known risk loci in this population (Fig. 2). Amish cases harbored a significantly higher burden of the known risk alleles compared to Amish cognitively normal controls (logistic regression, p = 1.01 x 10^–6^). As expected, the unrelated cases also had a significantly higher burden when compared to the unrelated cognitively normal controls (p < 2 x 10^–16^). When compared to unrelated cases, Amish cases had a significantly lower burden of known risk alleles (p = 1.60 x 10^–7^). Cognitively normal Amish controls were not different from the unrelated controls (p = 0.71). The difference between Amish LOAD cases (μ = 0.45 E4 risk alleles) and unrelated cases (μ = 0.82) was even greater when *APOE* was evaluated independent of the GWAS hits (p = 9.76 x 10^–8^).

### Case-control analysis of sequencing data

To focus further analysis on the variants most likely to contribute genetic risk to LOAD in the Amish, we sequenced 59 cases, 68 controls, and 49 unknowns most likely to harbor unidentified variants that confer risk to LOAD. The exomes sequenced harbored 155 variants in the known AD genes (Table 6). The most significant association p-value among these genes was 0.0098 for position 10,054,789 on chromosome 19 in *ABCA7*. This missense variant was not present in the three catalogs of human variation queried. Within the candidate linkage regions, 557 variants were identified and the most significant p-value was 0.00017 on chromosome 3 (Table 7). After correcting for the total number of variants tested in these three classes, no variant reached an experiment-wide level of significance.

**Table 6:** Summary of variants identified that are within or very near known AD genes.

Gene	Location	Variants	dbSNP	ESP	1000G	Novel
ABCA7*	19p13.3	20	19	18	18	1
***APOE***	19q13.2	0	0	0	0	0
***APP***	21q21.3	1	1	1	0	0
BIN1*	2q14	3	3	3	3	0
CASS4*	20q13.31	9	9	8	9	0
CD2AP*	6p12	1	1	1	1	0
CD33*	19q13.3	1	1	1	1	0
CELF1*	11p11	1	0	0	0	1
CLU*	8p21-p12	2	2	2	2	0
CR1*	1q32	9	9	9	9	0
DSG2*	18q12.1	8	7	7	5	1
EPHA1*	7q34	3	3	3	3	0
FERMT2*	14q22.1	5	4	4	4	1
HLA-DRB5/DRB1*	6p21.3	0	0	0	0	0
INPP5D*	2q37.1	2	2	2	2	0
MEF2C*	5q14	1	1	1	1	0
MS4A*	11q12.2	45	42	41	35	1
NME8*	7p14.1	0	0	0	0	0
PICALM*	11q14	2	2	2	2	0
***PSEN1***	14q24.3	1	1	1	1	0
***PSEN2***	1q31-q42	2	1	1	1	1
PTK2B*	8p21.1	12	10	11	10	1
SLC24A4/RIN3*	14q32.12	5	5	5	4	0
***SORL1***	11q23.2-q24.2	15	14	14	14	1
***TREM2***	6p21.1	1	1	1	1	0
ZCWPW1*	7q22.1	6	5	4	4	1

Counts are displayed for the number of variants present in the human variation catalogs of dbSNP build 137 (dbSNP), ESP 6500 release (ESP), and 1000 Genomes April 2012 release (1000G). The number of novel variants identified in each implicated gene is also shown.

* Closest gene to GWAS hit.

**Table 7:** Summary of variants identified within implicated linkage regions.

Peak	Variants	dbSNP	ESP	1000G	Novel
**Chr 2: 62–102 Mbp**	282	277	273	276	4
**Chr 3: 161–175 Mbp**	54	54	54	54	0
**Chr 9: 99–114 Mbp**	158	157	156	156	1
**Chr 18: 7–15 Mbp**	63	62	62	62	0

Counts are displayed for the number of variants present in the human variation catalogs of dbSNP build 137 (dbSNP), ESP 6500 release (ESP), and 1000 Genomes April 2012 release (1000G). The number of novel variants identified in each implicated linkage region is also shown. Chr = chromosome. Mbp = megabase pair.

In addition, and as a secondary screen, single variant case-control analysis was performed for all 79,203 exonic sequencing variants to test for possible association with LOAD in the Amish. The most significant p-value was 1.25 x 10^–6^ for positions 102,762,544 on chromosome 10 and 91,503,598 on chromosome 15. Thirteen additional exonic variants had p-values less than 1 x 10^–4^ (Table 8). None of these reach classical levels of genome-wide significance when corrected for multiple comparisons.

**Table 8:** MQLS-corrected allele frequencies and case-control association p-values for the top sequencing variants in the sequencing dataset.

Marker	Chr	Position	Case	Control	Overall	p value	Gene	Function
			MAF	MAF	MAF			
***rs41291476***	10	102762544	0.0104	0.0019	0.0013	1.25E-06	*LZTS2*	synonymous
***rs147224053***	15	91503598	0.0104	0.0019	0.0013	1.25E-06	*RCCD1*	synonymous
***rs4548***	3	128525253	0.0987	0.0259	0.0525	3.31E-06	*RAB7A*	synonymous
***rs11380***	12	6601475	0.0156	0.0078	0.0072	4.68E-06	*MRPL51*	missense
***rs201285308***	5	176008380	0.0156	0.0078	0.0072	5.00E-06	*CDHR2*	missense
***rs41279402***	20	3785672	0.0414	0.0036	0.0108	9.28E-06	*CDC25B*	UTR-3
**6_137234733**	6	137234733	0.0403	0.0171	0.0199	1.01E-05	*PEX7*	UTR-3
***rs11676272***	2	25141538	0.5938	0.4151	0.4619	1.28E-05	*ADCY3*	missense
***rs144407106***	6	136710582	0.0511	0.0203	0.0277	1.53E-05	*MAP7*	synonymous
***rs149872991***	15	91496233	0.0278	0.005	0.0085	3.74E-05	*UNC45A*	missense
***rs147643564***	4	175158508	0.0651	0.0195	0.0318	4.67E-05	*FBXO8*	UTR-3
***rs146399677***	20	3785297	0.041	0.0076	0.0129	4.89E-05	*CDC25B*	synonymous
***rs56400929***	10	105762909	0.0104	0	0.001	7.00E-05	*SLK*	missense
***rs150358287***	20	3687141	0.0894	0.0374	0.0455	7.94E-05	*SIGLEC1*	stop-gained
***rs34270879***	10	90673047	0	0.0434	0.0352	9.17E-05	*STAMBPL1*	missense

Chr = chromosome. MAF = minor allele frequency. Nucleotide position is based upon the UCSC hg19 human reference genome. Gene and Function annotated by SeattleSeq134.

### Case-control analysis of selected variants

To verify the sequence variants and to evaluate them in the full dataset, the 56 most significant candidate variants from the known AD genes and implicated linkage regions were genotyped and tested for association with LOAD in the Amish (S3 Table). No variant passed the significance threshold when corrected for 48 tests (p < 0.00104). The most significant result (p = 0.0012) was for *rs73938538* (MAF 0.087), a synonymous variant in *LAMA1* within the linkage peak on chromosome 18. No other variant was significant at a threshold of p < 0.05. Seven of the 48 markers had a p-value less than 0.1 (Table 9).

**Table 9:** MQLS-corrected allele frequencies and case-control association p-values for the top variants in the full dataset.

Marker	Chr	Position	Case MAF	Control MAF	p value	Gene
rs73938538*	18	7008583	0.1513	0.0766	0.0012	*LAMA1*
**11_47505996^+^**	11	47505996	0.0069	0.0001	0.0543	*CELF1*
rs1786263*	18	13116432	0.3063	0.3384	0.0550	*CEP192*
rs6505776*	18	12984144	0.3162	0.3423	0.0758	*SEH1L*
rs8244*	2	86371883	0.4026	0.4523	0.0775	*IMMT*
rs3772173*	3	170078232	0.1287	0.1635	0.0788	*SKIL*
***rs4811697*^+^**	20	55033856	0.3946	0.3700	0.0920	*CASS4*

Chr = chromosome. MAF = minor allele frequency. Nucleotide position is based upon the UCSC hg19 human reference genome. Gene annotated by SeattleSeq134.

* Variant in implicated linkage regions.

+ Variant in implicated AD gene.

To determine if the *rs73938538* association generalized in a dataset of unrelated cases and controls, the variant was genotyped in 473 LOAD affected individuals and 498 cognitively normal controls. When the variant was tested for association with LOAD, it failed to replicate (logistic regression with *APOE* as a covariate, p = 0.28). In this unrelated dataset, the minor allele frequency (MAF) in cases was 0.081 and 0.094 in controls, which is the opposite direction of effect of the minor allele in the Amish. This result was consistent in a large consortium-derived meta-analysis of 74,046 individuals investigating 7,055,881 genotyped and imputed SNPs (p = 0.21) [16].

## Discussion

The results of the genetic risk score analysis indicate that the common variants so far implicated by GWAS in European Caucasian populations explain a smaller proportion of genetic risk in the Amish than in the general population. The results of the genetic burden analysis of *APOE* only support the previous association of the E4 allele with LOAD in the Amish [26]. In the Amish from Elkhart, LaGrange and Holmes Counties, the *APOE* E4 allele has a frequency of 0.18 in cases, but in cases from the general Caucasian population this risk allele frequency is 0.42 [12,26]. This allele frequency disparity may in part explain the increase in difference in genetic burden between cases from the two datasets when only *APOE* was analyzed, but additional factors are likely to contribute as well. Although there is evidence that prevalence of dementia in the Amish may be somewhat lower than in the general population, the small sample size in these studies generates a very large confidence interval [30,31]. Our experience suggests that the prevalence is likely to be greater than these published reports. Further, these results indicate that genetic variation other than those already described is responsible for at least some of the Amish dementia.

Since Amish cases did have a higher burden when compared to cognitively normal controls from the same population, it can be assumed these known risk loci do explain some of the expected genetic effects. The concordance of the genetic risk scores between the general population cognitively normal controls and the Amish controls indicates that the Amish cognitively normal population is similar to their general population counterparts and is consistent with their shared ancestry.

A synonymous variant in *LAMA1*, *rs73938538*, is associated with LOAD in the Amish just below experiment-wide significance. While this association did not generalize in the unrelated dataset, a potential relationship between this variant and risk for LOAD is supported by the relevant function of this gene to LOAD pathophysiology. *LAMA1* encodes the laminin alpha subunit. Laminin is a major functional component of the basement membrane of many tissues, including the endothelium of blood vessel walls, and different isoforms may contribute to vascular homeostasis [32]. The alpha1 subunit of laminin is expressed in the basal lamina of blood vessels in the central nervous system, mostly confined to capillary walls [33]. There is strong evidence to suggest the etiology of LOAD may include cerebrovascular dysregulation and that the neuronal degeneration is secondary to this dysregulation [34,35]. This synonymous variant encodes for a more common valine codon (GTG) which has a frequency of 2.91 in highly expressed human genes and 2.78 in all human genes than the referent allele does (GTT) which has frequencies of 1.12 and 1.11, respectively [36,37]. The association of the synonymous variant *rs73938538* with LOAD in the Amish suggests that inefficient translation or abnormal co-translational folding of a protein important for cerebrovascular homeostasis and dysregulation may contribute to the underlying pathology and degeneration in this isolated population. This suggests *LAMA1* as a candidate gene for follow-up studies to further explore the relevance and consequences of the association of this variant and LOAD.

In the QCed sequence dataset comprised of 53 cases and 65 cognitively normal controls, the power to detect an association, if present, was limited by the allele frequency and the effect size of the variant. For a rare variant with a MAF of 1%, the probability of detecting an association was 90% if the OR was at least 26, an extremely large effect size. If the MAF was 3%, this study was at least 90% powered to detect an association if the OR was 11. If the variant was more common with a MAF of 5%, the sequence dataset was at least 90% powered to detect an association with a variant with an OR of 8. Because of this low power in the sequence dataset, the top candidate variants were selected for genotyping in the larger Amish dataset comprised of 126 cases and 503 controls. In this dataset, the study was at least 90% powered to detect an association with a variant that had a MAF of 1% and an OR of 8. It was at least 90% powered to detect an association if the MAF was 3% and the OR of the variant was 4. For a common variant with a MAF of 5%, the study was at least 90% powered to detect an association if the OR was 3.2. These power calculations are based upon a type I error rate of 0.05 and assume the samples are unrelated. The samples studied in the Amish datasets are related to one another, so these calculations are only estimates of the true power in these two datasets. Thus out data indicate that the Amish do not carry low frequency exonic variants contributing to their risk of dementia.

The lack of generalization in the unrelated dataset may be due to several reasons. First, the association detected in the Amish population may be a false positive and therefore not a true association. If this is true, the association should not be detected in any other study population or dataset. However, there was at least 90% probability to detect an association, if present in the unrelated dataset, if the true effect size is between 0.125 and 0.5 for the given sample size (n = 971) with a type I error of 0.05. Second, the association may true and detected because the Amish have a more homogeneous background. Third, the phenotype-genotype correlation may have arisen separately in the Amish after the founding of the population and could therefore be unique to this genetically isolated population. Fourth, the association with this variant and LOAD may be the result of an interaction with another genetic variation or a component of the environment that is unique to the Amish culture or way of life. If this interacting factor was untested or unaccounted for in this study, and therefore not reproduced in the unrelated dataset, the association may not be detected.

Six genes had multiple variants in the follow-up genotyping. In a large dataset, gene-burden analysis might be illuminating. However, the relatively small size of the Amish dataset and the low frequency of the observed variants conspire to severely underpower such an analysis.

The genetic risk score results suggest that the known LOAD risk loci explain a smaller proportion of the genetic risk in the Amish than in the general population. The targeted association results suggest that additional exonic variation in associated LOAD genes and regions implicated by previous linkage studies does not contribute risk to LOAD in the Amish, beyond the possible association with *LAMA1*. Other areas of the genome, intronic regulatory elements, epigenetic modifications, or previously unassociated genes, may be harboring variation that confers susceptibility in the Amish but were not interrogated by this study. Additional studies examining the non-exonic variation of known AD genes and the candidate linkage regions, as well as other portions of the genome, are likely to identify new variation that confers susceptibility to developing LOAD in the Amish.

## Supporting Information

S1 Table Details of 25 top variants identified from 26 known AD genes for follow-up genotyping. (XLSX)

S2 Table Details of 30 top variants identified from 4 implicated linkage regions for follow-up genotyping. (XLSX)

S3 Table Full Association Results for Follow-up Genotyping. (XLSX)
